# Structure-Guided Modification of *Rhizomucor miehei* Lipase for Production of Structured Lipids

**DOI:** 10.1371/journal.pone.0067892

**Published:** 2013-07-03

**Authors:** Jun-Hui Zhang, Yu-Yan Jiang, Ying Lin, Yu-Fei Sun, Sui-Ping Zheng, Shuang-Yan Han

**Affiliations:** Guangdong Key Laboratory of Fermentation and Enzyme Engineering, School of Bioscience and Bioengineering, South China University of Technology, Guangzhou, P. R. China; The Chinese University of Hong Kong, China

## Abstract

To improve the performance of yeast surface-displayed *Rhizomucor miehei* lipase (RML) in the production of human milk fat substitute (HMFS), we mutated amino acids in the lipase substrate-binding pocket based on protein hydrophobicity, to improve esterification activity. Five mutants: Asn87Ile, Asn87Ile/Asp91Val, His108Leu/Lys109Ile, Asp256Ile/His257Leu, and His108Leu/Lys109Ile/Asp256Ile/His257Leu were obtained and their hydrolytic and esterification activities were assayed. Using Discovery Studio 3.1 to build models and calculate the binding energy between lipase and substrates, compared to wild-type, the mutant Asp256Ile/His257Leu was found to have significantly lower energy when oleic acid (3.97 KJ/mol decrease) and tripalmitin (7.55 KJ/mol decrease) were substrates. This result was in accordance with the esterification activity of Asp256Ile/His257Leu (2.37-fold of wild-type). The four mutants were also evaluated for the production of HMFS in organic solvent and in a solvent-free system. Asp256Ile/His257Leu had an oleic acid incorporation of 28.27% for catalyzing tripalmitin and oleic acid, and 53.18% for the reaction of palm oil with oleic acid. The efficiency of Asp256Ile/His257Leu was 1.82-fold and 1.65-fold that of the wild-type enzyme for the two reactions. The oleic acid incorporation of Asp256Ile/His257Leu was similar to commercial Lipozyme RM IM for palm oil acidolysis with oleic acid. Yeast surface-displayed RML mutant Asp256Ile/His257Leu is a potential, economically feasible catalyst for the production of structured lipids.

## Introduction

Protein engineering or protein design is an important and effective strategy for developing biocatalysts. This strategy evolves or tailors enzymes to have desired properties such as higher enzyme activity, or better stability or selectivity [1], [2]. Structure-guided design, which takes advantage of known protein structure, is combined with *in silico* methods to select target amino acids for mutation. This avoids the time and labor of high-throughput screening from a library of thousands of mutants [3], [4].

Lipase esterification activity correlates with enzyme hydrophobicity to some extent. Increasing the hydrophobicity of a lipase can promote access of the substrate to the enzyme and can stabilize the substrate-enzyme complex by decreasing the binding energy in a nonaqueous phase [5]. In many cases, selection or modification of a lipase-immobilizing matrix or altering lipase regions to have increased hydrophobicity greatly enhances the lipase esterification activity [6]–[11]. In addition, directly modifying the lipase surface or lipase lid domain based on amino acid hydrophobicity increases esterification activity and can even change chain length selectivity [12]–[15]. Therefore, modifying the lipase-binding pocket using a structure-guided method to improve pocket hydrophobicity might increase lipase esterification activity.

Human milk fat (HMF) is mostly triacylglycerols, which are the main energy source in breast milk and infant milk formula. In typical HMF, the total fatty acid composition is about 21.8% palmitic acid and 33.9% oleic acid [16]. About 70% of the palmitic acid of HMF is in the sn-2 position; alternatively stated, 57.2% of the sn-2 position is occupied by palmitic acid [16]. The sn-1,3 position is mostly occupied by unsaturated fatty acids, mainly oleic acid (44%) followed by palmitic acid (18.7%) and stearic acid (14.2%) [17]. In fact, 1,3-dioleoyl-2-palmitoyl-glycerol (OPO, 19%) is the second most abundant triacylglycerol species in HMF [18]. OPO reduces the formation of calcium soaps, which causes stool hardness and constipation [19], [20]. *Rhizomucor miehei* lipase (RML) is a typical lipase that catalyzes the esterification or hydrolysis of lipids along with other oil modification reactions such as acidolysis, alcoholysis, glycerolysis and interesterification [21]. The crystal structure of RML has been solved [22]. With its good regioselectivity and relatively high incorporation rate, RML is an excellent lipase for the production of HMFS [23]. We developed a whole cell catalyst, a *Pichia pastoris* that displays RML on the cell surface, and used this catalyst for esterification of flavor esters and biodiesel production [24], [25]. Although yeast surface-displayed RML is inexpensive and recyclable, its esterification activity is still being developed.

In this study, we increased the hydrophobicity of the RML binding pocket based on a designed enzyme structure. We used computational software to model the mutated structure and evaluate the activity of mutant enzymes. These were tested for the production of HMFS in a solvent system with tripalmitin and oleic acid and in a solvent-free system with palm oil and oleic acid as substrates.

## Materials and Methods

### Chemicals

Yeast extract and tryptone were from OXOID (Basingstoke, UK). Peptone was from BD (Sparks, MD). Restriction enzymes, ligase and polymerase were from TAKARA (Dalian, China). *p*-nitrophenyl butyrate (pNPB) was from Sigma (St. Louis, MO). Organic acid, ethanol and other chemicals were analytical or higher grade. Tripalmitin was from Aladin (Shanghai, China) and palm oil was a gift from Shenzhen Jinyi Lipid Co. (Shenzhen, China). Commercial RML-lipozyme RM IM (*R. miehei* lipase immobilized on a macroporous anion-exchange resin) was from Novozyme (Tianjin, China).

### Strains and Media

*Escherichia coli* TOP10 and *Pichia pastoris* GS115 were from Invitrogen (USA). Strain GS115/pKFSR was *R. miehei* lipase displayed on the *P. pastoris* GS115 cell surface by flocculation using the Flo1p-short (FS) anchor protein [24].

Luria-Bertani medium (1% tryptone, 0.5% yeast extract, 1% NaCl), with ampicillin (100 µg/ml) added when necessary, was used to incubate and select *E. coli* transformants. YPD (1% yeast extract, 2% peptone, 2% dextrose) was used to incubate *P. pastoris* GS115 and maintain strains on agar plates. Yeast transformants were selected in MD medium (1.34% yeast nitrogen base [YNB], 400 µg/L biotin, 2% dextrose, 1.5% agar). *P. pastoris* was cultivated in BMGY (1% yeast extract, 2% peptone, 0.1 M potassium phosphate, pH 6.0, 1.34% YNB, 400 µg/L biotin, 1% glycerol) and BMMY (1% yeast extract, 2% peptone, 0.1 M potassium phosphate pH 6.0, 1.34% YNB, 400 µg/L biotin, 1% methanol).

### Mutant Construction

The overlap extension PCR method was used for site mutagenesis of *RML* with plasmid pKFSR [24] as template. RML was expressed with the surface-display anchor protein on the N-terminus and a Flag tag on the C-terminus.

Primer sequences were:

RML-F: 5′- CGC GGC ACG CGT GTT CCA ATT AAG AGA CAA TCT AACT -3′, RML-R: 5′-GCC AGC GAA TTC TTA CTT ATC GTC GTC ATC CTT GTA ATC AGT ACA CAA ACC AGT GTT AAT ACC -3′, with underlining showing restriction sites *Mlu*I and *Eco*RI respectively. N87I-F: 5′-ATT AGA *ATC* TGG ATT GCT GAT TTG ACT-3′, N87I-R: 5′- AGT CAA ATC AGC AAT CCA *GAT* TCT AAT -3′, N87I/D91V-F: 5′- ATT AGA *ATC* TGG ATT GCT *GTT* TTG ACT-3′, N87I/D91V-R: 5′- AGT CAA *AAC* AGC AAT CCA *GAT* TCT AAT -3′, H108L/K109I-F: 5′- AAG GTT *CTT ATC* GGT TTT TTGG -3′, H108L/K109I-R: 5′- CCA AAA AAC C*GA TAA G*AA CCTT -3′, D256I/H257L-F: 5′- CTG TTT TG*A TCC TT*T TGT CTT AC -3′, D256I/H257L-R: 5′- GTA AGA CAA *AAG GAT* CAA AAC AG -3′, with italic letters showing mutation sites.

The *RML* gene was amplified with *Mlu*I and *Eco*RI restriction sites on both ends, and inserted into digested vector pKFS. The mutated pKFSR plasmids were used to transform *E. coli* TOP10 for amplification and then linearized and used to transform *P. pastoris* GS115.

The *RML* gene in *P. pastoris* GS115 was amplified with the KOD FX PCR system (Toyobo, Shanghai, China) directly from colonies and sequenced by BGI (Guangzhou, China) to verify mutations.

### Computational Analysis of Mutants

Discovery Studio V3.1 (Accelrys, San Diego, CA) was used for computational analysis of mutants. Structures were created by the protocol Macromolecules-Protein Design-Build Mutants. Binding energy was calculated by the protocol Receptor-Ligand Interaction-CDocker.

### RML Expression

*P. pastoris* strains were inoculated into 50 ml of BMGY medium in a 500 ml flask. After 24 h of cultivation, the culture was centrifuged at 5000 rpm for 6 min and the pellet was resuspended in BMMY medium containing 1% methanol at an OD_600_ of 1. Methanol was added every 24 h to the culture to a final concentration of 1%. Cells were incubated for 6 days at 30°C with shaking at 250 rpm.

Cells displaying RML were centrifuged at 5000 rpm for 6 min, washed with water, and centrifuged again. Pellets were resuspended in 4 ml 50 mM Tris-Cl buffer (pH 8.0) and 0.5 ml 10% (*m/v*) trehalose and lyophilized (Christ Beta 1–8, Germany).

### Enzyme Activity Assay

Hydrolytic activity was determined colorimetrically using pNPB as substrate. Cell-displayed lipases were dissolved in lipase assay buffer (50 mM Tris-HCl, pH 8.0), mixed with substrate solution (0.0625 mM pNPB, 0.1% Triton X-100 in lipase assay buffer), and reacted at 45°C for 5 min before measuring OD_405_ with a kinetic microplate reader. Reactions were terminated by 20 µl 20% trichloroacetic acid. After centrifugation at 10,000×*g* for 1 min, a 200 µl aliquot of supernatant was placed in a 96-well plate and measured. Product yields were determined from a standard curve using *p*-nitrophenol as a standard. One unit of hydrolytic activity was defined as the amount of enzyme that released 1 µmol *p*-nitrophenol from substrate per minute.

Synthetic activity was determined by the synthetic reaction of ethanol with organic acid such as caprylic acid, capric acid, lauric acid, or oleic acid. Substrates composition were 0.25 M ethanol and 0.2 M acid with heptane as solvent. Yeast displaying RML in lyophilized powder form (100 mg) were suspended in 5 ml substrate mixture in a 25 ml conical flask, stoppered to prevent evaporation of solvents and reactants. Reactions were carried out at 55°C at 200 rpm in an orbital shaker. After reactions, solutions were centrifuged at 12,000 rpm for 1 min. Supernatant was titrated with 0.02 M sodium hydroxide to determine the amount of residual acid. One unit of synthetic activity was defined as the amount of enzyme that synthesized 1 µmol of ethyl ester per minute.

### Flow Cytometry Analysis

Yeast cells were harvested by centrifugation after methanol induction for 120 h. Immunofluorescence analysis was performed using a modified version of the Kobori et al. protocol [26]. Yeast cells were resuspended in phosphate-buffered saline (PBS, pH 7.4) supplemented with 1% (*m/v*) bovine serum albumin (BSA) to block the cell surface. A monoclonal antibody (Agilent, Santa Clara, CA), against FLAG tag (DYKDDDDK) was used as the primary antibody. The cell suspension (200 µl, OD_600_ = 1) was incubated with 1 µl of antibody (2 µg/µl) at room temperature for 2 h. Cells were washed twice with PBS and resuspended in 200 µl PBS (with 1% BSA), before being exposed to the secondary antibody (1 µl, 2 µg/µl) Alexa Fluor 488 conjugated goat-anti-mouse IgG (Invitrogen, Grand Island, NY), for 1 h at room temperature. Cells were washed three times with PBS. Part of the cell suspension was used for flow cytometry analysis (Quanta SC, Beckman Coulter). Each sample was counted for 10,000 cells and analyzed with Exp032 software (Beckman Coulter) to obtain the total and average fluorescence value.

### Lipase Catalyzed Acidolysis

Tripalmitin and oleic acid in organic solvent: Acidolysis reactions were 120 mg tripalmitin and 240 mg oleic acid (minimum purity 85%), dissolved in 6 ml heptane. Either whole cell RML catalyst or commercial RML-Lipozyme RM IM was used at 54 mg. The mixture was placed in 25 ml Erlenmeyer shaking flasks for incubation in a water bath shaker at 200 rpm, 55°C. Samples were withdrawn from the reaction mixture at predetermined time intervals for analysis.

Palm oil and oleic acid in solvent-free system: Acidolysis reactions used 3 g palm oil, 6 g oleic acid, 0.9 g lipase without solvent. The mixture was placed in 50 ml Erlenmeyer shaking flasks for incubation in a water bath with magnetic agitation at 200 rpm, 55°C. Samples were taken from the reaction mixture at predetermined time intervals for analysis.

All reactions were performed at least in triplicate, and means were used to evaluate results.

### Fatty Acid Assay

Triglyceride isolation: Samples were isolated by thin-layer chromatography (TLC, silicic acid 60G, thickness 1 mm, 20 cm×20 cm) with petroleum ether, diethyl ether, and acetic acid (90∶10∶0.5, v/v/v) as developing solvent [27]. Bands containing triacylglycerols (TAGs) were removed by scraping and extracted with isooctane. Isolated TAGs were used to analyze the fatty acid compositions.

Methylation and fatty acid (FA) composition analysis by gas chromatography (GC): Bands corresponding to TAGs were removed by scraping and methylated to fatty acid methyl esters (FAME). Samples were saponified with 0.5 M NaOH in methanol (4 ml) at 80°C for 5 min, and methylated with 14% BF_3_ in methanol (5 ml) for 10 min. After cooling to room temperature, isooctane (3 ml) and saturated NaCl were added and mixed, and the upper isooctane layer was collected. An Agilent 7890A gas chromatograph equipped with a hydrogen flame-ionization detector and a DB-FFAP silica capillary column (0.25 mm×30 m, Agilent, Santa Clara, CA, USA) was used to analyze fatty acid composition. The injector and detector were set at 250°C. The carrier gas was nitrogen, and the total gas flow rate was 23 ml/min. Injector temperatures was 250 °C and detector temperature was 260 °C. Column temperature was 150 °C for 3 min increasing to 215 °C for 10 min at the rate of 10 °C/min. A sample of 1 µl was injected, and the FAME content was calculated using C17∶0 as an internal standard.

## Results and Discussion

### Selection of Amino Acids for Mutation

The catalytic triad of RML is formed by Ser144, His257 and Asp203. The RML lid is a 15-amino acid helix from residues 82 to 96, in which residues 83–84 and 91–95 are hinges. Opening the lid exposes the binding pocket, which has the shape of a shallow bowl [28].

**Figure 1:**
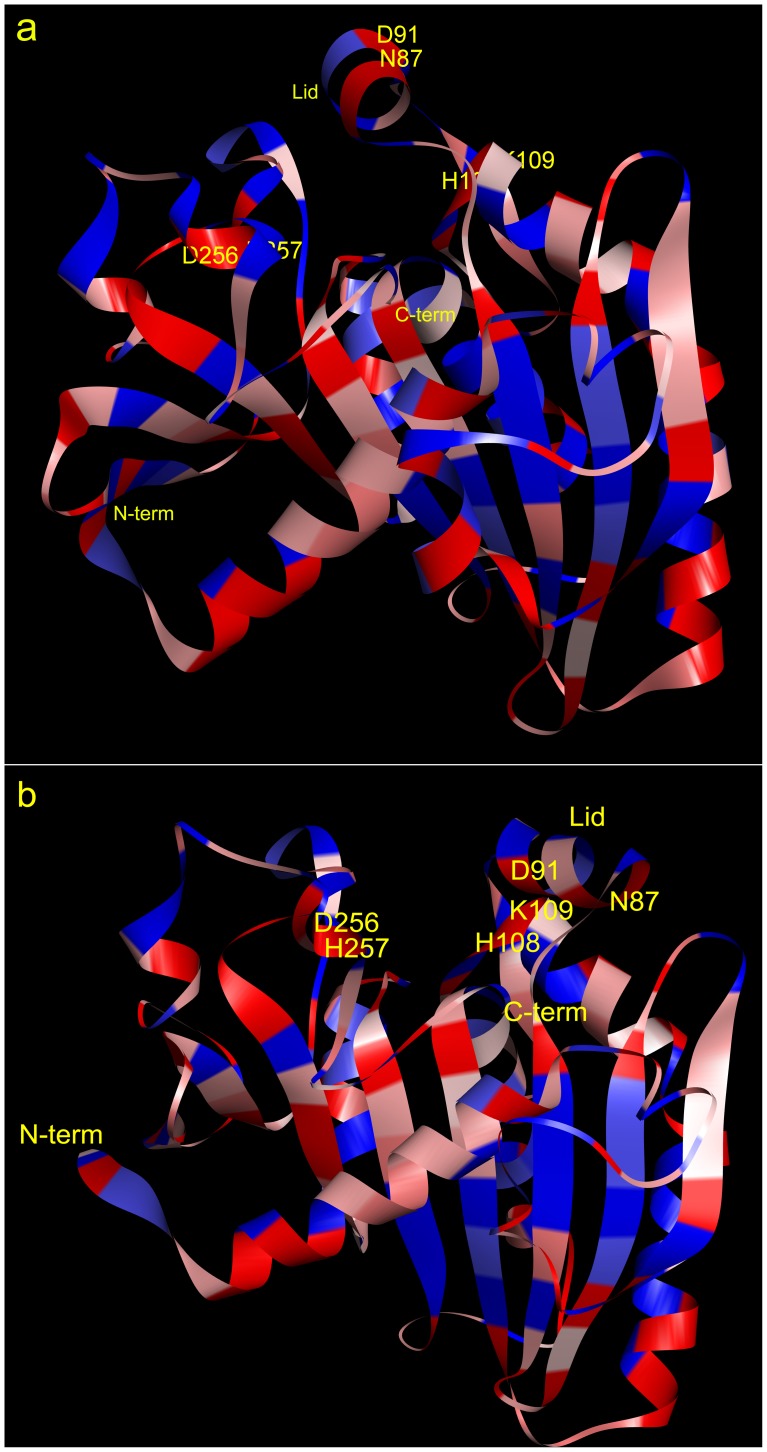
Amino acid hydrophobicity profile of RML. a: 3TGL, lid-closed form, b: 4TGL, lid-open form. Red, hydrophilic residues; blue, hydrophobic residues.

From Protein Data Bank (PDB), we obtained the open structure (PDB ID: 4TGL) and the closed structure (PDB ID: 3TGL) of RML. In the binding pocket and the lid (Fig. 1), Asn87 (−3.5), Asp91 (−3.5), His108 (−3.2), Lys109 (−3.9), Asp256 (−3.5) and His257 (−3.2) are exposed on the inner side of the binding pocket and have low hydrophobic values (listed in the bracket after the residue). To increase the hydrophobicity of the binding pocket, we mutated the above residues to Val (4.2), Leu (3.8) and Ile (4.5), which have higher hydrophobic values [29].

We organized the six residues into three groups according to their positions [30]. Considering that Asn87 was on the lid helix and Asp91 was in the hinge area, we mutated Asn87Ile/Asp91Val and Asn87Ile alone to avoid influencing lid movement. Since His108-Lys109 and Asp256-His257 were adjacent residues, we mutated both residues simultaneously to His108Leu/Lys109Ile and Asp256Ile/His257Leu.

### Computational Analysis of Mutants

**Figure 2:**
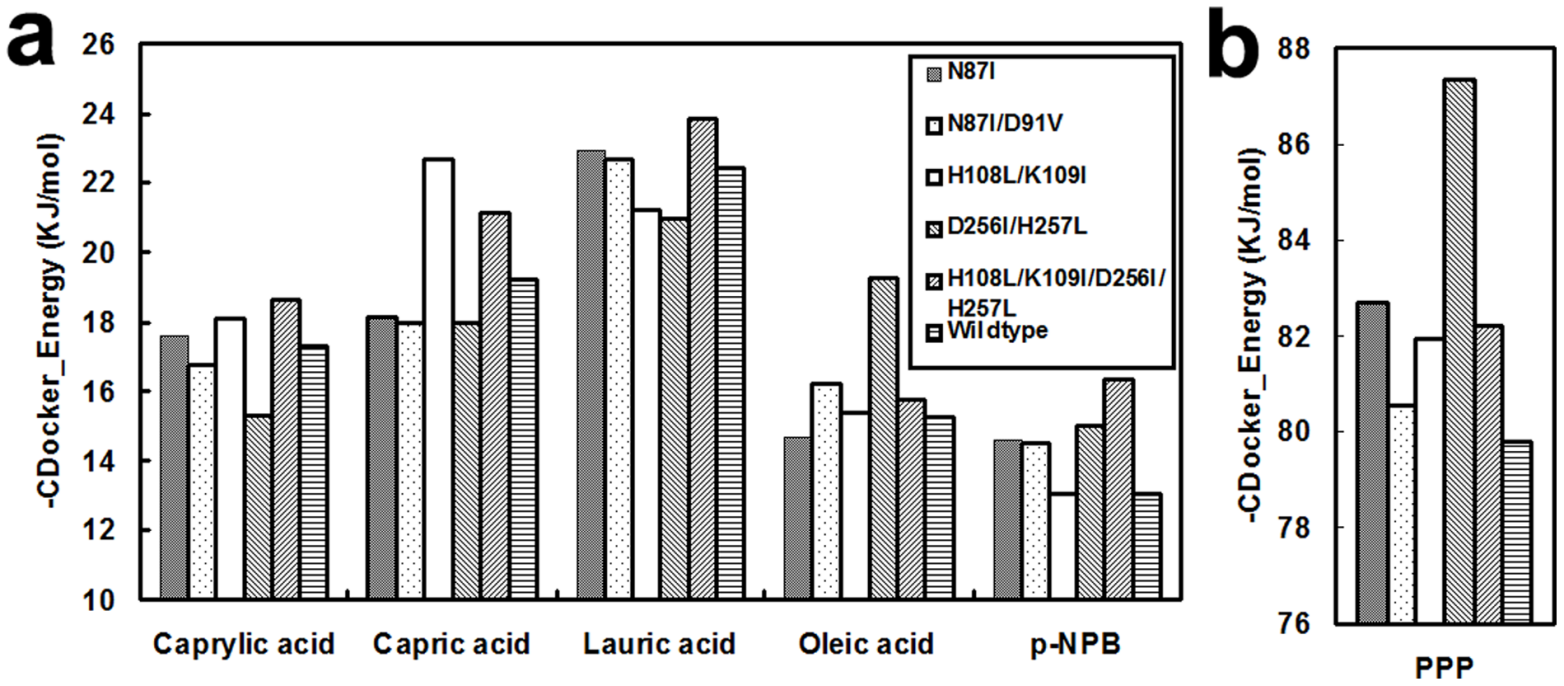
CDocker energy of RML mutants and wild-type with different substrates. a and b are separate because of discrepancies in the range of their Y-axes. a and b have the same legend. For comparison with the enzyme activity data, the energy value was converted to positive.

Discovery Studio 3.1 was used to simulate the protein structures of five RML mutants: Asn87Ile, Asn87Ile/Asp91Val, His108Leu/Lys109Ile, Asp256Ile/His257Leu, His108Leu/Lys109Ile/Asp256Ile/His257Leu. From the structures, we selected the one with the lowest energy to dock with hydrolytic and esterification substrates (Fig. 2). CDocker is a semi-flexible docking program in which the ligand is flexible. CDocker searched ligand configurations randomly using a dynamic method, and configurations in the active site of the enzyme were optimized by simulated annealing.

The results showed that the CD_docker energy of the mutants with most substrates was not always lower than wild-type. However, for oleic acid, pNPB and tripalmitin (PPP), several mutants had significantly lower CD_docker energy than wild-type. We assayed enzyme activity in addition to the computation analysis.

### Enzyme Activity

**Figure 3:**
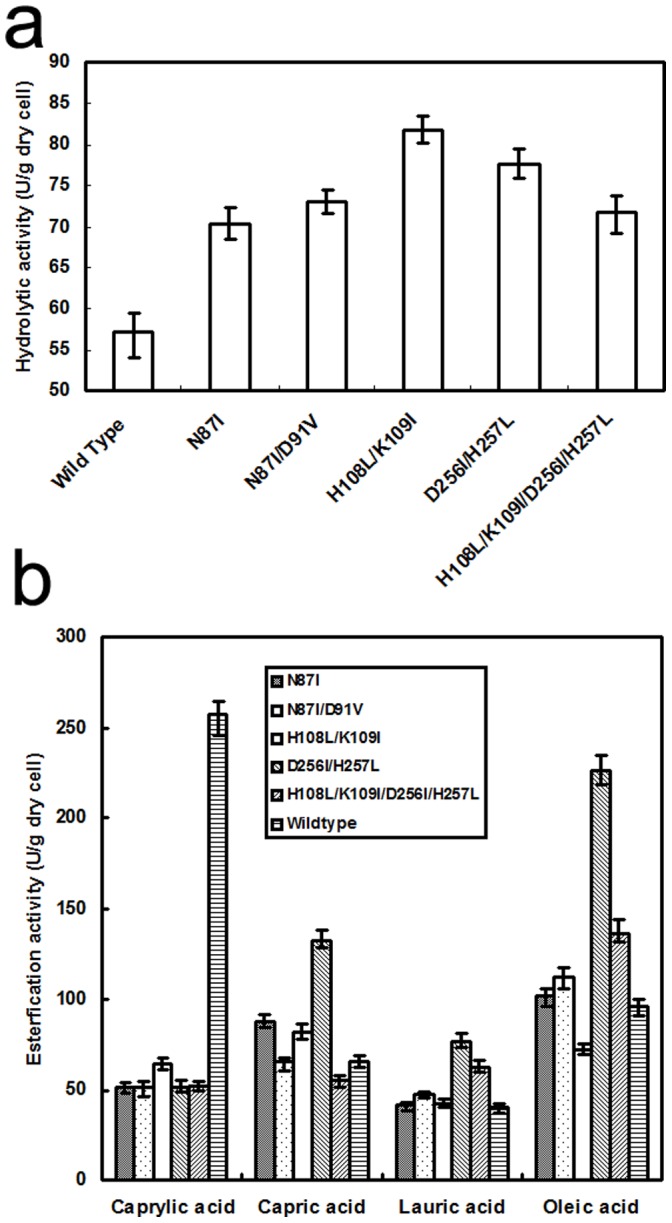
Flow cytometry of whole cell RML catalysts. a: Asn87Ile/Asp91Val, b: His108Leu/Lys109Ile, c: Asp256Ile/His257Leu, d: His108Leu/Lys109Ile/Asp256Ile/His257Leu, e: wild-type, f: negative control.

To determine whether the variation in enzyme activity was caused by the mutant structure or a change in the level of expression on the cell surface, we used flow cytometry to evaluate cell-surface fluorescence. The average fluorescence in relative fluorescence units was 475.32 for cells displaying Asn87Ile/Asp91Val, 461.09 for His108Leu/Lys109Ile, 483.88 for Asp256Ile/His257Leu, 476.10 for His108Leu/Lys109Ile/Asp256Ile/His257Leu, and 466.74 for wild-type (Fig. 3). Liang et al. demonstrated a linear relationship between recombinant yeast cell fluorescence and the number of the enzyme proteins on the cell surface [31]. Taking account of the systematic error in our method, the fluorescence of the mutants and wild-type showed no obvious difference. This result indicated the same offset value as the negative control. Therefore, we concluded that expression of the mutants and wild-type RML was within the same order of magnitude.

**Figure 4:**
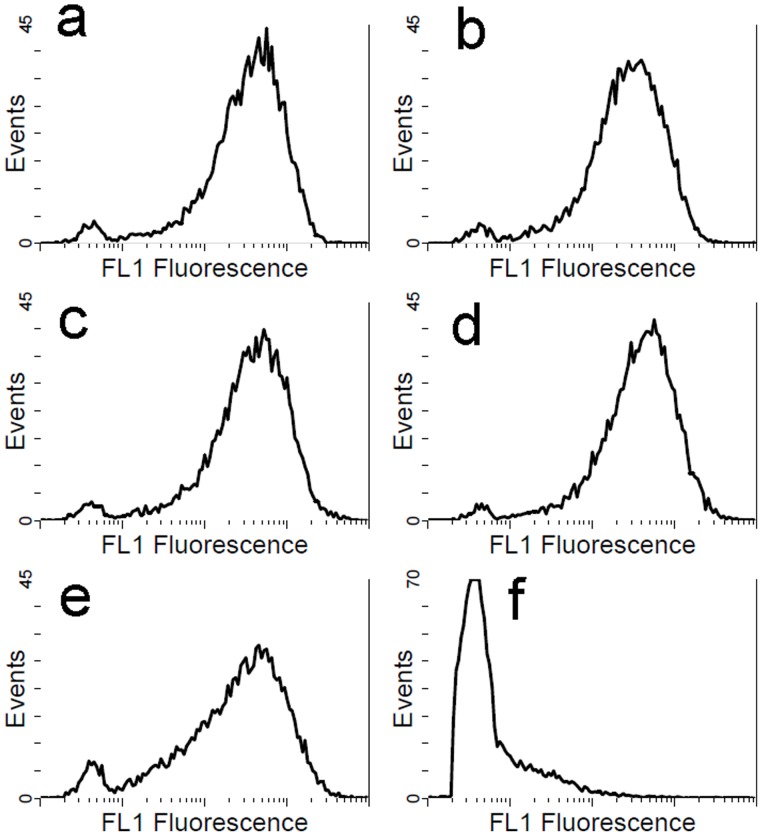
Enzyme activity of RML wild-type and mutants. a: hydrolytic activity, b: esterification activity.

The hydrolytic activity of the five mutants and wild-type RML were assayed (Fig. 4a). The hydrolytic activity was 70.2 U/g for yeast displaying Asn87Ile, 73.1 U/g for Asn87Ile/Asp91Val, 81.8 U/g for His108Leu/Lys109Ile, 77.6 U/g for Asp256Ile/His257Leu, 71.7 U/g for His108Leu/Lys109Ile/Asp256Ile/His257Leu and 57.1 U/g for wild-type RML. Activity for all mutants was higher than for wild-type RML, and among them the mutants His108Leu/Lys109Ile and Asp256Ile/His257Leu showed the greatest enhancements of 43.2% and 36.0% compared to wild-type.

Esterification activity was also determined according to a synthesis reaction with ethanol and several organic acids with different carbon chain lengths from C8 to C18∶1 (Fig. 4b). For caprylic acid, the wild-type activity was greater than for the mutants; mutant activity was about 34% of wild-type activity. For the other acids however, most mutant activities were higher than the wild-type. The activity of Asp256Ile/His257Leu was significantly higher than wild-type when reacting with three acids, but not caprylic acid.

Among the five mutants, His108Leu/Lys109Ile had the highest hydrolytic activity and Asp256Ile/His257Leu had the highest esterification activity. Mutants Asn87Ile and Asn87Ile/Asp91Val showed higher hydrolytic and esterification activity (except caprylic acid as substrate) than wild-type, but less than Asp256Ile/His257Leu. His108, Lys109, Asp256 and His257 are all located in the binding pocket but not the lid. This suggested that mutation of the binding pocket changed the enzyme characteristics. However, the His108Leu/Lys109Ile/Asp256Ile/His257Leu mutation did not show a synergetic effect (Fig. 4). In fact, the hydrolytic and esterification activities of the quadruple mutant were higher than wild-type, but lower than the double mutant Asp256Ile/His257Leu.

### Comparison with Computational Analysis

Although the enzyme activity did not completely match the values from CDocker_Energy, we drew some conclusions from the results.

For most substrates, the binding energy of mutants was decreased compared to the wild-type enzyme, indicating that the enzyme activity might be increased (Fig. 2). However, for caprylic acid, enzyme activity of all the mutants was reduced (Fig. 4b). For other substrates, the activity of the quadruple mutant might be higher than the double mutants by predicted docking energy, but the actual esterification activity of the quadruple mutant was lower than the double mutant Asp256Ile/His257Leu (Fig. 4b). The reason for this result might be that the protein structure in organic media was different from the crystal structure, or that the program accuracy was decreased for predicting the results of additional mutations [32]. The prediction also showed that mutation of additional residue of His108Leu/Lys109Ile did not add to the effect of Asp256Ile/His257Leu on the esterification activity. The docking energy of Asn87Ile was predicted to be lower than Asn87Ile/Asp91Val, which predicted that the mutation Asp91Val in the hinge might hinder binding of the substrate in the pocket (Fig. 2).

Mutation in the binding pocket affected acyl carbon chain-length specificity in esterification by RML. In our case, the change in specificity resulting from hydrophobicity variance maybe relate to three factors. First, hydrophobic interactions between the mutants and substrates are different. A change in hydrophobicity in binding pocket influences the orientation and degree of binding of the substrate. Second, the size of the alcohol and acyl groups affects the approachability of the binding pocket; this might result in changes in selectivity for the optimal acid. Next, mutation of the lid or hinge influences the opening and closing of the lid. Santarossa et al. found that mutations in the *Pseudomonas fragi* lipase lid region influence chain-length specificity of the substrate [14]. The relative activity of C8/C4 increased with mutation of one, two, or three residues, but the ratio of C12/C4 was not related to the number of mutations. Discovery Studio and its predecessor Insight II are powerful tools in drug design and enzyme engineering. However, the software accuracy is not perfect, resulting in discrepancies between the activity of some mutants and the docking results [32].

For oleic acid and tripalmitin (PPP), which are the two substrates for HMFS modification, the mutant Asp256Ile/His257Leu showed a distinct decrease in docking energy compared to the wild-type and the other mutants, predicting a significant increase in enzyme activity (Fig. 2). The prediction was verified by the esterification of ethanol and oleic acid (Fig. 4b). Based on the measured enzyme activity and the predicted docking energy, the four mutants including Asn87Ile/Asp91Val, His108Leu/Lys109Ile, Asp256Ile/His257Leu, and His108Leu/Lys109Ile/Asp256Ile/His257Leu were used for OPO synthesis and oil modification for HMFS production.

### Whole Cell Catalysts in HMFS Modification

For the production of structured lipids, TAG acidolysis with fatty acids catalyzed by 1,3-specific lipase was a useful strategy. The process was TAG hydrolysis, then DAG or MAG esterification with acid with possible acyl immigration from the 1,3-position to the 2-position of glycerol [17]. The hydrolytic and esterification activity of the lipase were both important for product yield. When lipase esterification activity was increased, the incorporation of acid and the structured lipid yield increased.

**Figure 5:**
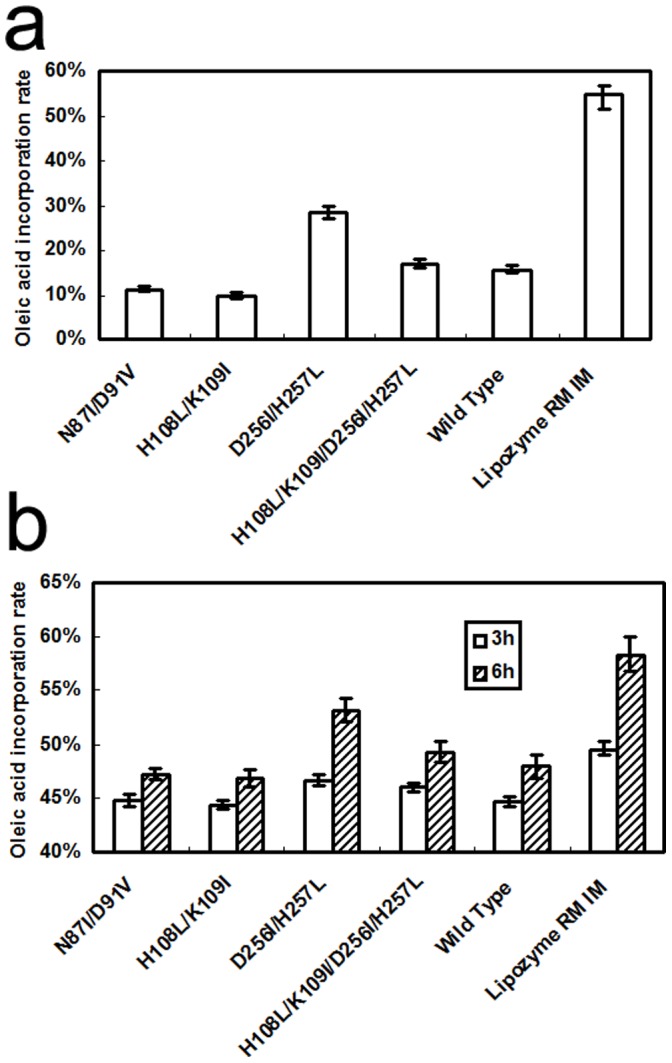
OA incorporation of whole cell catalyst and commercial lipase. a: OA incorporation in a reaction of PPP with OA for 24 h; b: OA incorporation in a reaction of palm oil with OA for 3 h and 6 h.

We first evaluated the performance of the mutants for synthesizing structured lipid-OPO in organic solvent with tripalmitin and oleic acid as substrates, with cell-displayed wild-type RML and commercial RML-Lipozyme RM IM as controls. After 24 h of reaction, oleic acid incorporation was 15.57% for wild-type, 11.93% for Asn87Ile/Asp91Val, 9.65% for His108Leu/Lys109Ile, 28.27% for Asp256Ile/His257Leu, and 16.94% for His108Leu/Lys109Ile/Asp256Ile/His257Leu (Fig. 5a). Incorporation did not increase for all mutants compared to the wild-type. Of four of the mutants, the incorporation of Asp256Ile/His257Leu was the highest, at 1.82-fold the wild-type level. This incorporation was close to the result of Tecelão [33], which was 32.1% at 24 h. The incorporation of Asn87Ile/Asp91Val and His108Leu/Lys109Ile were decreased 23.4% and 38.0% relative to the wild-type. The quadruple mutant showed an increase of 8.8% of the wild-type rate. This result coincided with the Discovery Studio prediction for Asp256Ile/His257Leu and the quadruple mutant (Fig. 2).

Increasing the hydrophobicity of the lipase or the immobilizing support was a feasible way to improve lipase esterification activity. Our results also indicated a difference in oleic acid (OA) incorporation by the mutant with the highest activity, Asp256Ile/His257Leu, and Lipozyme RM IM (Fig. 5). Immobilization was an effective way to increasing RML stability and activity [11], [34], [35]. Lipozyme RM IM carrier, an anion-exchange resin, has hydrophobic properties and disperses well in solvent, contributing to esterification activity [36]. Hydrophobicity might be a reason for differences in activity between yeast-displayed RML and Lipozyme RM IM.

### Production of HMFS in a Solvent-free System

Oil acidolysis with OA was a viable and useful way to simplify HMFS production. We used cell-displayed RML wild-type and mutants for acidolysis palm oil with oleic acid in a solvent-free system. OA incorporation was 47.11% for Asn87Ile/Asp91Val, 46.80% for His108Leu/Lys109Ile, 49.22% for His108Leu/Lys109Ile/Asp256Ile/His257Leu, and 47.99% for wild-type (Fig. 5b). Asp256Ile/His257Leu showed excellent performance with an oleic acid incorporation of 53.18% after 6 h. This incorporation was similar to reports from Esteban and Telecão [16], [33]. Esteban had an oleic acid incorporation of about 48% after 6 h in a reaction of a palmitic acid-rich TAG with an oleic acid-rich free fatty acid (FFA) catalyzed by Lipozyme RM IM [16]. Telecão had an OA incorporation of about 41.5% after 48 h in a reaction of tripalmitin with oleic acid catalyzed by *Carica papaya* lipase extracted from petiole leaves [33].

Oleic acid is about 40% in the fatty acids of initial palm oil. Compared to palm oil substrate, the increase in incorporation of oleic acid was 7.11% by Asn87Ile/Asp91Val, 6.80% by His108Leu/Lys109Ile, 13.18% by Asp256Ile/His257Leu, 9.22% by His108Leu/Lys109Ile/Asp256Ile/His257Leu and 7.99% by wild-type. In this reaction system, the catalysis efficiency of Asp256Ile/His257Leu was the highest at 1.65-fold of wild-type.

Of note, Asp256Ile/His257Leu showed an oleic acid incorporation of 53.18%, which was close to the 58.32% of Lipozyme RM IM when palm oil and oleic acid were the substrates.

### Hydrophobicity and Enzyme Activity

In our study, we increased the hydrophobicity of the lipase substrate-binding pocket; this was a small area compared to the entire cell-catalyst surface. In fact, other approaches to changing the hydrophobicity of whole cells such as displaying hydrophobin on the yeast surface together with the lipase, might also increase esterification activity, since yeast cells act as the matrix to immobilize the enzyme. A combination of traditional immobilization technology with protein structure design, specifically immobilizing the enzyme after site-directed mutagenesis on the hydrophobic support, could be a powerful way to improve enzyme activity [37].

However, an increase in hydrophobicity does not always result in enhancement of esterification activity [38]. In addition to the hydrophobicity, open or closed enzyme form; lipase type, specifically active center conformation; solvent polarity or ion strength; and substrate molecular size or chain length are all important factors that affect lipase esterification activity. For immobilized enzymes, the kind of immobilizing support; the immobilization pattern; the presence of detergents; and the immobilization strategy, such determining as the ion strength or pH of solutions, can all influence lipase form, structure, and rigidity, affecting activity or selectivity [35].

Even in cases in which hydrophobicity enhances enzyme activity, the mechanisms are complex and different. One of the most important mechanisms is activation of lipases in the presence of a hydrophobic support interface to form a lid-open state [7], [9], [35]. In addition, using an immobilized carrier could lower the retention of water or acids, which also contributes to improved activity. The hydrophobicity of the carrier helps the disperse the enzyme in the substrate [36]. Modification of lid hydrophobicity balanced the hydrophobic/hydrophilic structure of the lid in the environment [12]. Mutations on lipase lid increased hydrophobic and electrostatic interactions between the lid and substrate [15].

Compared with previous reports, we found that modification of the binding pocket improved substrate-enzyme interaction. Docking results showed that docking energy decreased after mutation. However, simply promoting binding was not the only factor in the activity increase, because binding with the active center or recognition center, or reversible or irreversible binding had different consequences. Lid movement is the essential factor affecting enzyme activity of lipases with lids. Our results suggested that lid movement might be changed in mutations, since activity of Asn87Ile and Asn87Ile/Asp91Val were lower than Asp256Ile/His257Leu (Fig. 4).

**Figure 6:**
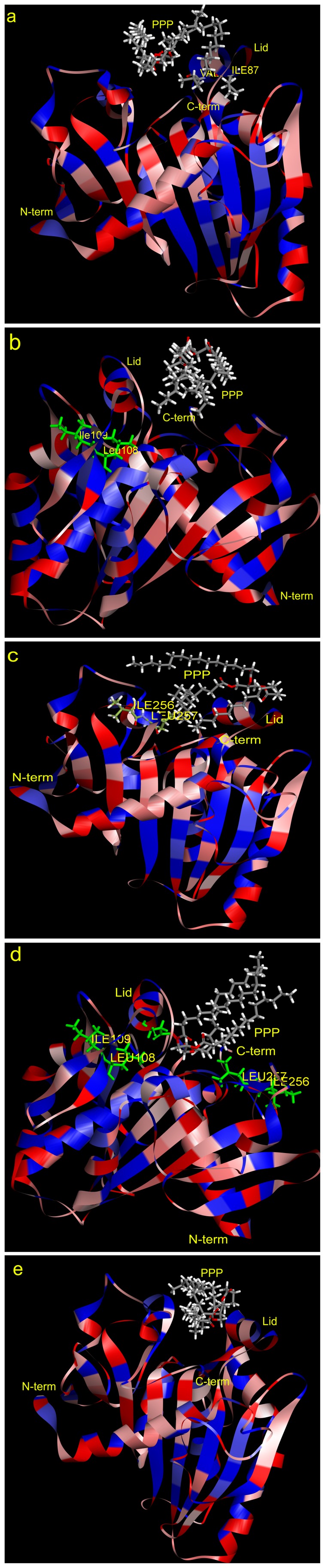
Profiles of RML mutants and wild-type docking with PPP. a: Asn87Ile/Asp91Val, b: His108Leu/Lys109Ile, c: Asp256Ile/His257Leu, d: His108Leu/Lys109Ile/Asp256Ile/His257Leu, e: wild-type.

The pattern and orientation of RML mutants docking with PPP were different (Fig. 6). For Asn87Ile/Asp91Val, His108Leu/Lys109Ile and wild-type, PPP reacted with lipase in a horizontal orientation and with a shallower entry of the substrate into the pocket than Asp256Ile/His257Leu and His108Leu/Lys109Ile/Asp256Ile/His257Leu. His108Leu is located at the bottom of the binding pocket. In His108Leu/Lys109Ile/Asp256Ile/His257Leu, an alcohol moiety entered the pocket and the acyl part protruded. Asp256Ile/His257Leu is located on the opposite side of the lid, which caused the acyl moiety to be closer to the binding pocket. The three acyl chains separated farther than the other mutants. Since Leu255 and Leu258 are both hydrophobic amino acids, when connected to Asp256Ile/His257Leu, the binding pocket was most suitable for long-chain substrates. These factors meant that Asp256Ile/His257Leu had higher activity than the other mutants in this study.

### Conclusion

We found that, with the same enzyme load, displayed RML had an OA incorporation comparable to Lipozyme RM IM for HMFS production. The whole-cell catalyst has a simpler productive technology and a higher expression level. As an enzyme modified based on hydrophobicity, the yeast surface-displayed RML mutant Asp256Ile/His257Leu is a promising catalyst for HMFS production, and could be a potential alternative catalyst for other oil modifications.
