# Correction: Structure-Guided Modification of *Rhizomucor miehei* Lipase for Production of Structured Lipids

**DOI:** 10.1371/annotation/e4b16300-84fa-4c3b-97fe-4eb8fe881d00

**Published:** 2013-10-24

**Authors:** Jun-Hui Zhang, Yu-Yan Jiang, Ying Lin, Yu-Fei Sun, Sui-Ping Zheng, Shuang-Yan Han

Figures 3 and 4 were incorrectly switched. The image currently appearing as Figure 3 belongs with the title and legend for Figure 4, and the image currently appearing as Figure 4 belongs with the title and legend for Figure 3. The titles and legends are in correct order. 

